# Crystal and electronic structure of PbTe/CdTe nanostructures

**DOI:** 10.1186/1556-276X-6-126

**Published:** 2011-02-10

**Authors:** Małgorzata Bukała, Piotr Sankowski, Ryszard Buczko, Perła Kacman

**Affiliations:** 1 Institute of Physics PAS, Al. Lotnikow 32/46, 02-668 Warsaw, Poland; 2 Institute of Informatics, University of Warsaw, St. Banacha 2, 02-097 Warsaw, Poland

## Abstract

In this article, the authors reported a theoretical study of structural and electronic properties of PbTe inclusions in CdTe matrix as well as CdTe nano-clusters in PbTe matrix. The structural properties are studied by *ab initio *methods. A *tight-binding *model is constructed to calculate the electron density of states (DOS) of the systems. In contrast to the *ab initio *methods, the latter allows studying nanostructures with diameters comparable to the real ones. The calculations show that both kinds of inclusions lead to changes of the DOS of the carriers near the Fermi level, which may affect optical, electrical and thermoelectric properties of the material. These changes depend on the size, shape, and concentration of inclusions.

## Introduction

PbTe is a well-known narrow-gap semiconductor. This material is widely used for mid-infrared lasers and detectors [1,2]. Moreover, PbTe has attracted a lot of interest due to its thermoelectric properties, and the material is used for small-scale cooling applications as well as for power generation in remote areas [3,4]. The efficiency of a thermoelectric device is described by the dimensionless thermoelectric figure-of-merit parameter *ZT*. In the currently used thermoelectric devices based on PbTe, Si-Ge, or Bi_2_Te_3 _alloys, *ZT *reaches 1. This value imposes limitation to possible applications of semiconductor thermoelectric devices, and a lot of effort is put to increase the parameter.

Increased *ZT *values were observed in various low dimensional nanostructures, like quantum wells or coupled semiconductor quantum dot (QD) systems of PbTe or Bi_2_Te_3 _[5-7]. These observations were explained by the fact that introducing defects or nano-inclusions, i.e. creating materials with nanometer-scaled morphology reduces dramatically the thermal conductivity by scattering phonons. In nanostructures composed of canonical thermoelectric materials, an increase of the *ZT *parameter is also expected, because the qualitative changes of electronic density of states (DOS) in quantum wells, wires, and dots should increase the Seebeck coefficient. Indeed, new materials with improved electronic and thermal properties were obtained by an enhancement of DOS in the vicinity of the Fermi level. In Ref. [8], an enhancement of thermoelectric efficiency of PbTe by distortion of the electronic DOS using thallium impurity levels was reported.

The studies of pseudo-binary alloys consisting of PbTe inclusions in CdTe matrix started with the discovery of sharp PbTe-CdTe superlattices [9]. PbTe and CdTe have nearly the same cubic lattice constant *a*_0_: 0.646 and 0.648 nm, respectively. It should be recalled that lead telluride crystallizes in rock-salt (RS) structure while cadmium telluride crystallizes in zinc-blende (ZB) structure. The materials can be represented by the two, cation and anion, interpenetrating fcc sub-lattices. In both cases, the cation sub-lattice is shifted with respect to the Te anion sub-lattice along the body diagonal [1, 1, 1]; in the RS structure it is shifted by *a*_0_/2, whereas in the ZB structure by *a*_0_/4. Nanometer-sized clusters (QDs) of PbTe in CdTe matrix were obtained by a proper choice of the MBE-growth temperature and/or post-growth thermal treatment conditions [10,11]. Such system, which consists of QDs of a narrow energy gap material in wider gap matrix, is excellent for infrared optoelectronic applications. Careful theoretical studies of the interfaces between PbTe dots and CdTe matrix were reported in Ref. [12-15]. These structures are not conducting and seem to be of no thermoelectric relevance. However, chains of PbTe QDs or PbTe quantum wires (NWs) embedded in a CdTe matrix can have interesting thermoelectric properties. Recently, it was also shown that nanometer-sized clusters of wide-gap CdTe in narrow-gap PbTe matrix, which will be called quantum anti-dots (A-QD), can be obtained and can lead to a considerable increase of the thermoelectric figure-of-merit parameter *ZT *[16].

In this article, a systematic theoretical study of PbTe-CdTe pseudo-binary systems is presented. Using *ab initio *and *tight-binding *methods, three kinds of inclusions are studied: the PbTe NWs in CdTe matrix; the CdTe A-QDs; and anti-wires (A-NWs) in PbTe matrix. The aim of this research is to check how introducing nanostructures of different size and shape changes DOS of the carriers near the Fermi level.

## Model nanostructures and calculation method

**Figure 1:**
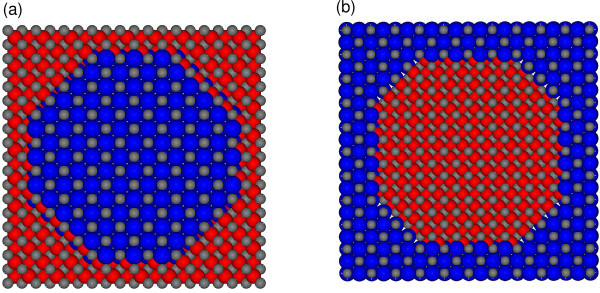
**(Color online) Cross section of the supercell of (a) RS PbTe NW in ZB CdTe matrix, (b) ZB CdTe A-NW in RS PbTe matrix**. The blue, red, and grey balls denote Pb, Cd, and Te atoms, respectively.

**Figure 2:**
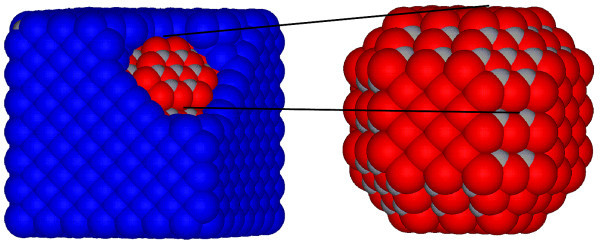
**(Color online) Model of a CdTe A-QD embedded in a PbTe matrix**. The blue, red, and grey balls denote Pb, Cd, and Te atoms, respectively. The whole rhombo-cubo-octahedral A-QD is shown in the inset.

The model nano-objects are cut out from the bulk material: the NWs from PbTe, whereas A-NWs and A-QDs from CdTe. The considered nano-objects are then inserted into the matrix composed of the other material, assuming common Te sub-lattice. In the calculations, periodic boundary conditions are used. The interfaces between the NWs (A-NWs) and the matrix are of {110} and {001} type. The same two types of planes and the {111} planes form the interfaces of the A-QD. As shown already in Ref. [12], the energies of all these interfaces are comparable, and the shape of 3 D nano-objects, from Wulff construction, should be rhombo-cubo-octahedral (the shape of the cross section of the wires should be a regular octagon). Cross-sectional views of the exemplary supercells of the NW and A-NW considered, are presented in Figure 1. In Figure 2, model of CdTe A-QD embedded in PbTe matrix is shown. The sizes of the simple-cubic supercells vary with the diameter of the nano-objects and the distances between them, i.e. with the thickness of the material of the matrix, which separates the inclusions. Our NWs and A-NWs are directed along the [001] axis and have diameters ranging from 1.2 to 10 nm. The considered A-QDs have diameters up to 4 nm. The distances between these inclusions are ranging from 0.6 to 2.6 nm.

For nanostructures, containing less than 500 atoms in the unit cell, all the atomic positions are calculated using the first principles methods based on the density functional theory, with full relaxation and re-bonding allowed. *Ab initio *calculations are performed with the *Vienna ab initio simulation package *[17,18]. For the atomic cores, the projector-augmented wave pseudo-potentials [19] are used. The exchange correlation energy is calculated using the local density approximation. The atomic coordinates are relaxed with a conjugate gradient technique. The criterion that the maximum force is smaller than 0.01 eV/Å is used to determine equilibrium configurations. Since the impact of nonscalar relativistic effects on the structural features is negligible [12,20], these effects are not taken into account.

The obtained relaxed structures are further used in the calculations of electron DOS, which are performed within the *tight-binding approximation*. We use the combined, *ab initio *and *tight-binding*, approach because calculating the DOS by first principles is very time consuming and does not lead to proper values of the energy gaps. The time of *tight-binding *calculations scales considerably slower with the number of atoms in the studied objects, and this method allows studying structures with more realistic dimensions.

Both materials, CdTe and PbTe, are described using the *sp*^3 ^atomic orbitals, with the interactions between the nearest neighbours and the spin-orbit coupling (SOC) included. The empirical *tight-binding *parameters for CdTe, which lead to proper values of the energy gaps and effective masses in the valence and conduction bands, are taken after Ref. [21]. For PbTe, it was verified that the *tight-binding *parameters available in the literature [22,23] do not lead to the effective masses determined experimentally. Thus, a new parameterization of PbTe bulk crystal was performed, which gives not only proper energy values at the important band extremes but also proper values of the longitudinal and perpendicular effective masses at the L point of the Brillouin zone. The details of this parameterization will be presented elsewhere.

To study the PbTe/CdTe systems, the knowledge of the band offsets between these two materials is needed. Since the valence band maxima of PbTe and CdTe are located at different positions in *k*-space, the valence band offset (VBO) can only be directly accessed in experiments allowing for indirect transitions, i.e. in experiments with momentum transfer to the electrons. However, in many experiments, e.g. in zero-phonon photoluminescence measurements or optical absorption spectra, only direct transitions are allowed. In such cases, local band offsets at certain *k*-points have to be considered, which are in general larger than the global band offsets [24]. The VBO of PbTe/CdTe (111) heterojunction interface was experimentally determined in Refs. [25,26]. In Ref. [25], the value of VBO Δ*E*_V _= 0.135 ± 0.05 eV was obtained using X-ray photoelectron spectroscopy. On the other hand, in Ref. [26], the VBO value Δ*E*_V _= 0.09 ± 0.12 eV was determined from the ultraviolet photoelectron spectrum using synchrotron radiation. Theoretically, the VBOs for PbTe/CdTe (100) and (110) interfaces were obtained by Leitsmann et al. [24,27]. The reported value of the VBO for polar PbTe/CdTe (100) interface is 0.37 ev, and it is 0.42 eV for the nonpolar PbTe/CdTe (110) interface. These values were obtained without the SOC. Adding the spin-orbit interaction diminished the VBO nearly to zero. Because of the large spread of these values and because experimental data are determined with very big errors, it has been decided to obtain the VBO by another *ab initio *procedure. Using a model of nonpolar (110) PbTe/CdTe interface, first the projected densities of states (PDOS) for two different Te atoms, both situated far from the interface (one in PbTe and the second in CdTe material) are calculated. In this calculation, the spin-orbit interactions were taken into account, because the electronic properties of PbTe are largely influenced by SOC [24]. Next, the densities of the deep d-states of the Te atom far from the interface with the Te atom in the bulk material are compared, also with SOC included. The above comparison is performed both for PbTe and CdTe. It is observed that each of the obtained PDOS is shifted in energy relative to PDOS of Te atoms in the bulk material. The sum of these differences gives us the VBO between PbTe and CdTe, which is equal to 0.19 eV.

Another problem, which needs to be solved, is related to the *tight-binding *description of the Te ions at the interfaces. The relevant integrals between the Te and Cd states are simply taken equal to those in CdTe. Similarly, the integrals between Pb and Te are assumed to be like in PbTe. The integrals are scaled with the square of the distances between the atoms and with the directional cosines. The problem appears when the energy values for *s *and *p *states of Te have to be chosen--they can be equal to the energies of Te either in CdTe or in PbTe. They can also be somehow weighted by taking into account the number of appropriate neighbours. In our study of the two-dimensional PbTe/CdTe heterostructures, all the three possibilities have been checked. It is observed that taking the energies of Te like in PbTe is the only way to avoid interface states in the PbTe band gap. Since experimentally these states have not been observed, in the following the Te atoms in the interface region are treated like atoms in PbTe.

The DOS is calculated near the top of the valence band (in *p*-type) or the bottom of the conduction band (in *n*-type). To check how introducing nanostructures of different size and shape changes DOS of the carriers near the Fermi level, the results have to be compared with the DOS for bulk material. In all the studied structures the same carrier concentration *n *(or *p*) = 10^19 ^cm^-3 ^is assumed. The energy zero is always put at the resulting Fermi level. As the total DOS depends on the size of the supercell, it should be normalized to the number of atoms. It was checked, however, that the DOS in the vicinity of the Fermi level in the PbTe/CdTe structures is equal to the DOS projected on the atoms in PbTe region. This means that, near the Fermi level, the DOS in the studied structures is determined by the states of electrons localized in PbTe. Thus, the DOS of these structures is normalized to the number of atoms in PbTe region only.

## Results

**Figure 3:**
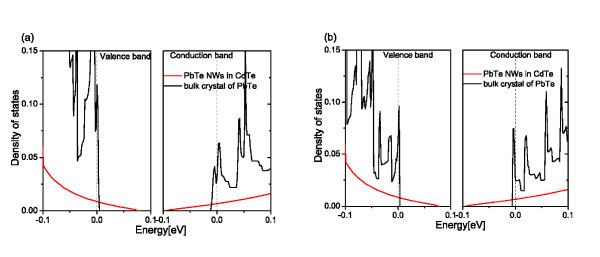
**(Color online) The DOS near the Fermi level for PbTe NW in CdTe matrix (black line) with not-relaxed (a) and relaxed (b) atomic positions**. The diameter of the wire is 3.6 nm. Here, and in all following figures, the energy zero in the valence and conduction bands was put at the energy corresponding to Fermi level for carrier concentration *p*(*n*) = 10^19 ^cm^-3^. The red lines refer to the bulk crystal of PbTe.

**Figure 4:**
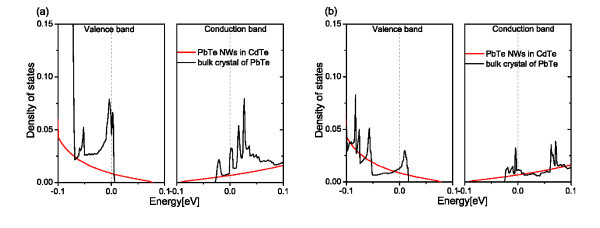
**(Color online) The DOS near the Fermi level for PbTe wires in CdTe matrix with not-relaxed atomic positions**. The wire diameters are 5 nm **(a) **and 9 nm **(b)**.

In Figure 3, the difference in DOS for PbTe NWs of diameter about 3.6 nm with relaxed and not-relaxed atomic positions is presented. It can be observed that, for such a small structure, the relaxation changes DOS but its qualitative character remains the same. As the *ab initio *computations are highly time consuming, the DOS for structures containing more than 500 atoms, has been calculated without relaxation of the atomic positions. The role of the relaxation, which proceeds mainly at interfaces, should diminish with the size of the structure. The long-range stress relaxation is omitted in the *tight-binding *calculations, due to the very good match of the PbTe and CdTe lattice constants. In Figure 4 the calculated DOS of PbTe NWs in CdTe matrix with not relaxed atomic positions for larger diameters is presented. In both Figures 3 and 4, it can be noticed that quantum confinement of PbTe wires leads to 1 D sub-bands and abrupt changes of the carrier DOS with energy. Thus, the derivative of the DOS at the Fermi level depends strongly on its position, i.e. on carrier concentration--small changes of the latter can lead even to a sign change in the derivative. As the energy spacing between the 1 D sub-bands depends on the confinement potential, the DOS depends strongly on the diameter of the NWs, as shown in the figures.

**Figure 5:**
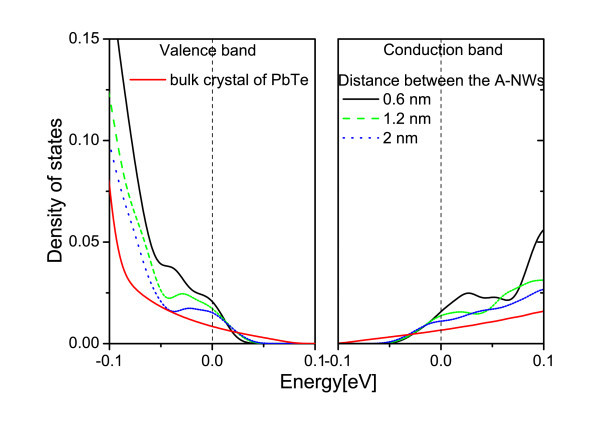
**(Color online) PbTe matrix with 6-nm-thick CdTe A-NWs**. The DOS near the Fermi level for the distance between the wires equal: 0.6 nm (black line), 1.2 nm (dashed green line), and 2 nm (dotted blue line).

**Figure 6:**
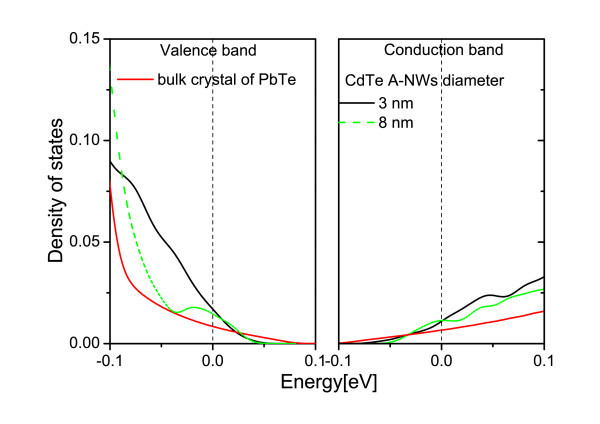
**(Color online) The DOS near the Fermi level for PbTe matrix with CdTe A-NWs**. The distance between the A-NWs is always the same, 1.2 nm. The diameters of the A-NWs are 3 nm (black line) and 8 nm (dashed green line).

**Figure 7:**
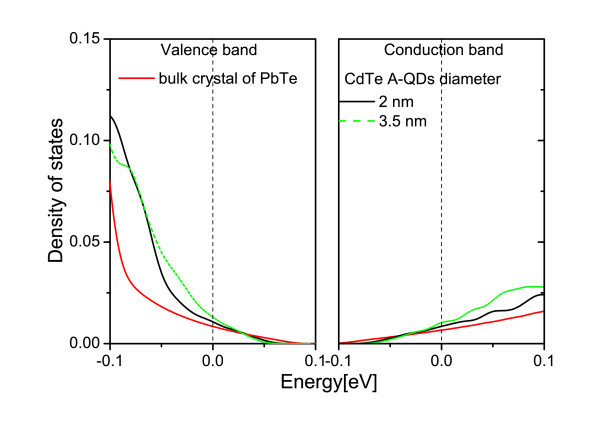
**(Color online) The DOS near the Fermi level for PbTe matrix with CdTe A-QDs**. The diameters of the A-QDs are 2 nm (black line) and 3.5 nm (dashed green line). The distance between the A-QDs is always the same, 1.2 nm.

Next, ZB CdTe A-NWs and A-QDs embedded in RS PbTe matrix are described. It can be recalled that in contrast to the NWs, in the anti-structures, the carriers are located in the PbTe channels between inclusions and can move in any direction. Thus, the low-dimensional sub-bands in the DOS are not to be expected. Still, how the DOS changes with the diameter of the anti-objects and the thickness of the PbTe matrix between the inclusion walls is studied. At first, the distance between the model A-NWs is changed while their diameter is kept constant. The results are presented in Figure 5. One notes that the thicker the PbTe channels between A-NWs, the less the DOS differs from that of PbTe bulk material. Diminishing the distance between the A-NWs leads to an increase of the DOS derivative at the Fermi level for both kinds of carriers. In Figure 6, the results for different diameters of A-NWs separated by the same distance are presented. The resonances in the DOS, which can be seen in the figure, result most probably from the confinement in the PbTe material in-between CdTe A-NWs. These PbTe channels can be considered as interconnected NWs. In Figure 7, similar results obtained for A-QDs, with diameters 2 and 3.5 nm, are shown. In the case of A-QDs, there is much more PbTe material in-between the inclusions, as compared to the A-NWs, and here the resonances are less pronounced and appear for higher energies.

## Conclusions

Using *ab initio *and *tight-binding *methods, the DOS for three kinds of PbTe-CdTe pseudo-binary systems is studied, i.e. PbTe NWs embedded in CdTe matrix; the CdTe A-QDs; and A-NWs in PbTe matrix. The results of our calculations show that quantum confinement of PbTe wires leads to 1 D sub-bands and changes dramatically the derivative of the electron DOS at the Fermi level. In the case of CdTe anti-inclusions (A-NWs and A-QDs), the DOS of carriers in PbTe matrix depends on both the diameter and the concentration of the anti-inclusions. This study shows that both kinds of inclusions, i.e. RS PbTe clusters in ZB CdTe matrix and CdTe nano-clusters in PbTe, lead to considerable changes of the derivative of the carrier DOS at the Fermi level and thus, can influence the thermoelectrical properties of the material. For PbTe NWs the changes are, however, very abrupt and sensitive to the carrier concentration. Thus, it seems that the anti-structures are much more suitable for controlled design.

## Abbreviations

DOS: density of states; NW: nanowire; PDOS: projected densities of states; QD: quantum dot; RS: rock-salt; SOC: spin-orbit coupling; VBO: valence band offset; ZB: zinc-blende.

## Competing interests

The authors declare that they have no competing interests.

## Autors' contributions

MB carried out the *ab initio *and *tight-binding *calculations, participated in data analysis and drafted the manuscript. PS made the *tight-binding *parameterization. RB and PK conceived of the study, participated in its design and coordination, analyzed and interpreted data, and wrote the manuscript. All authors read and approved the final manuscript.
